# MScanFit motor unit number estimation of abductor pollicis brevis: Findings from different experimental parameters

**DOI:** 10.3389/fnagi.2022.953173

**Published:** 2022-10-17

**Authors:** Ya Zong, Zhiyuan Lu, Peipei Xu, Maoqi Chen, Lianfu Deng, Sheng Li, Yingchun Zhang, Qing Xie, Ping Zhou

**Affiliations:** ^1^Department of Rehabilitation Sciences, Ruijin Hospital, Shanghai Jiao Tong University School of Medicine, Shanghai, China; ^2^School of Rehabilitation Science and Engineering, University of Health and Rehabilitation Sciences, Qingdao, China; ^3^Shanghai Key Laboratory for Prevention and Treatment of Bone and Joint Diseases, Department of Orthopaedics, Ruijin Hospital, Shanghai Institute of Traumatology and Orthopaedics, Shanghai Jiao Tong University School of Medicine, Shanghai, China; ^4^Department of Physical Medicine and Rehabilitation, University of Texas Health Science Center at Houston, and TIRR Memorial Herman Hospital, Houston, TX, United States; ^5^Department of Biomedical Engineering, University of Houston, Houston, TX, United States

**Keywords:** compound muscle action potential (CMAP), CMAP scan, motor unit number estimation (MUNE), MScanFit, abductor pollicis brevis (APB)

## Abstract

MScanFit motor unit number estimation (MUNE) based on the recording of the compound muscle action potential (CMAP) scan has wide applications. This study evaluated the effect of different CMAP scan settings on MScanFit MUNE. CMAP scan of the abductor pollicis brevis (APB) muscle was performed in 10 healthy subjects at a United States (US) research center using different stimulus pulse widths (0.1, 0.2 ms) and total number of stimuli or steps (500, 1,000), and in 12 healthy subjects at a China research center using a 0.1 ms pulse width and 500 steps. MScanFit MUNE was derived using the default model parameters. A significantly higher MUNE was obtained using the shorter than longer pulse width; 84.70 ± 21.56 (500 steps) and 77.90 ± 27.62 (1,000 steps) at a pulse width of 0.1 ms vs. 67.60 ± 18.72 (500 steps) and 62.20 ± 15.82 (1,000 steps) at a pulse width of 0.2 ms (*p* < 0.05). However, MUNE was unrelated to the number of steps (500 vs. 1,000, *p* > 0.1). MUNE was significantly higher in persons studied in the China center (136.42 ± 32.46) than the US center (84.70 ± 21.56) despite each center using the same pulse widths and steps (*p* < 0.001). After excluding the ethnicity, age and experimenter factors, this significant difference is speculated to be partly related to different electrode size used in the two centers. The findings suggest that CMAP scan experimental parameters should remain consistent, so the MScanFit MUNE will not be compromised by non-physiological factors.

## Introduction

Motor unit number estimation (MUNE) provides an important biomarker for examination of neuromuscular diseases and healthy aging. Most MUNE methods are based on sampling relatively low threshold units (de Carvalho et al., 2018). The recently developed MScanFit MUNE approach (Bostock, 2016; Jacobsen et al., 2018a) has the advantage of sampling both low and high threshold units through the recording of finely graded stimulus-response curve, i.e., compound muscle action potential (CMAP) scan (Visser and Blok, 2009). This approach may provide a more representative indication of motor unit size and thus MUNE. In this protocol, a large number of CMAPs are recorded in response to repetitive transcutaneous motor nerve stimulation at the full range of stimulus intensities. The MScanFit program models the recorded CMAP to estimate the number of motor units by minimizing the discrepancy between the modeled and experimental scan. Another attractive feature of this approach is that both CMAP scan recording and modeling are quick. This makes the MScanFit program a convenient tool for examining or tracking neuromuscular diseases.

When performing a CMAP scan, different protocols or settings can be used such as stimulus pulse width, the number of stimuli (steps), and stimulation frequency. Different pulse widths, steps and stimulation frequencies have been used in CMAP scan studies, revealing that there is no general agreement on a standardized protocol. By varying stimulus pulse widths (0.1, 0.2 ms), steps (500, 1,000) and stimulation frequencies (1, 2, 3 Hz), Maathuis et al. (2012) found thenar and hypothenar CMAP scan properties were most influenced by pulse width and number of steps, but not stimulation frequency. By varying stimulus pulse widths (0.1, 0.2, 0.6, and 1.0 ms), Sleutjes et al. (2021) found it significantly affected motor unit threshold distribution and alternation within thenar CMAP scans. In a previous study, we have examined the influence of different stimulation protocols on MScanFit of the first dorsal interroseous (FDI) for the ulnar nerve and found that stimulus pulse width, but not the number of steps, had a significant effect on MUNE (Zong et al., 2020). In the current study we set to further investigate the effect of different CMAP scan stimulus protocols on MScanFit MUNE of the abductor pollicis brevis (APB) innervated by the median nerve. Given that both stimulus pulse width and number of steps can influence CMAP scan variables (Maathuis et al., 2012), they may potentially affect MScanFit MUNE. Therefore, a combination of two of the most often used pulses widths (0.1, 0.2 ms) and steps (500, 1,000) was tested in the present study. Since stimulation frequency has been shown to have little effect on CMAP scan variables (Maathuis et al., 2012), a fixed stimulation frequency was used in this study.

As an emerging technique with quick and automated implementation, MScanFit MUNE has been used widely in different research and clinical centers. However, comparison of normative data from different centers is still lacking. Therefore, in addition to examining different stimulus protocols, we also compared MScanFit MUNE performance in two research centers using the same stimulus protocol and modeling procedures, and discuss potential factors that may influence MUNE. The findings from different CMAP scan protocols and different centers may help better understand factors that impact MScanFit MUNE, thus facilitating its appropriate application and interpretation in research and clinical practice.

## Methods

### Subjects

Ten neurologically intact subjects (5 male and 5 female) participated in the study at the University of Texas Health Science Center at Houston (UTHealth) and TIRR Memorial Hermann Hospital (Houston, TX). Their mean age was 33.60 ± 8.14 years and height was 167.70 ± 8.59 cm. Eight subjects were right handed and two were left-handed. The protocol was approved by the Committee for Protection of Human Subjects (CPHS) at UTHealth and TIRR Memorial Hermann Hospital.

Twelve neurologically intact subjects (7 male and 5 female) participated in the study in Ruijing Hospital, Shanghai Jiao Tong University School of Medicine (SJTU). Their mean age was 26.75 ± 1.67 years and height was 170.42 ± 8.70 cm. Nine subjects were right handed and three were left-handed. The protocol was approved by SJTU’s Institutional Review Board.

For both research sites, all participants gave written informed consent. Standard electrodiagnostic conduction studies of the median nerve were performed for each subject to exclude carpal tunnel syndrome. All the subjects showed that the latency, amplitude, and conduction velocity were in the normal range.

### Experiment

#### Experimental setup

**FIGURE 1:**
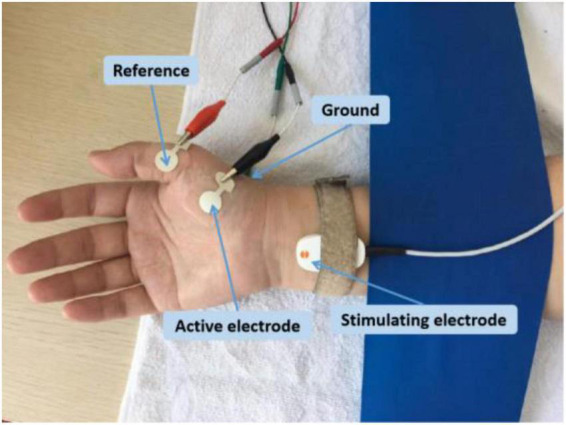
Electrode placement for CMAP scan recording of the APB.

The experimental setup was similar in both research sites. Each subject was comfortably seated in a chair with the forearm relaxed on a height-adjustable table. The APB muscle in the dominant side was examined. The skin temperature was maintained above 32°C. The examined hand was restrained in supination by Nylatex^®^ wraps in order to minimize movement artifacts. The skin was cleaned with alcohol pads before applying the electrodes. Subjects were asked to remain completely relaxed during the recording. The active electrode was placed on the motor point of the APB muscle, and the reference electrode was placed on the metacarpal phalangeal joint of the thumb (Figure 1). A self-adhesive electrode was placed on the dorsum of the hand as the ground electrode. A standard bar electrode (9 mm diameter for each of the two contact surfaces, 20 mm apart), placed 1–2 cm proximal to the wrist (cathode distal), was used to activate the median nerve. The electrode was coated with conductive paste. Initially, the optimal stimulus site was found by shifting the electrode position to result in a large CMAP at low stimulus intensity. Once this position was determined, the electrode was secured in place with surgical tape and self-adherent wrap.

At UTHealth, all the data were collected using an UltraPro S100 EMG system (Natus Neurology Incorporated, Middleton, WI, USA). The CMAP was recorded using disposable Ag–AgCl surface electrodes (10 mm diameter, button electrode). At SJTU, all the data were collected using a Nicolet EDX EMG system (Natus Neurology Incorporated). CMAP was recorded using disposable Ag–AgCl surface electrodes (13 mm diameter, plus extended portion for connecting with alligator clip, Figure 1).

#### Compound muscle action potential scan

For both research sites, the CMAP scan was recorded using the built-in program of the clinical EMG machine. A pre-scan program was run first to search for the upper and lower stimulation current intensities. The current was finely adjusted to determine the lowest threshold that evoked a response (i.e., single motor unit or groups of motor units). Subsequently, the current progressively increased until the maximum CMAP was elicited. To account for the baseline noise and response variability, the upper stimulation intensity (S100) was set 1 mA above the highest threshold and the lower stimulation intensity (S0) was 2 mA below the lowest threshold. The stimulus intensity range was defined as the difference between the upper and lower intensities.

The CMAP scan was carried out after setting the intensity parameters. Stimulation parameters including the stimulation frequency, pulse width, range of intensity, and number of steps were configured in the user interface. Stimuli were delivered repeatedly at 2 Hz, with a linearly declining intensity from the upper to the lower intensity. The program stopped after the stimulus intensity reached the lower limit. In the UTHealth site, a combination of two different steps (500, 1,000) and two different pulse widths (0.1 ms, 0.2 ms) was carried out on each subject for CMAP scan recording. The order of the different stimulation protocols was randomized for each subject. In the SJTU site, a protocol of 500 steps and pulse width of 0.1 ms was used for CMAP scan recording. To avoid possible confounding effects of muscle fatigue, adequate rest was provided between recordings.

### MScanFit

The free version of the MScanFit program (version 10/8/2015) (Bostock, 2016) was used to estimate motor unit number in both UTHealth and SJTU sites. Firstly, prescan (encompassing the baseline EMG) and postscan (encompassing repeated maximal CMAPs) time periods of the CMAP scan were set by the user in order to estimate the signal variance within these periods. Subsequently, a preliminary model was set, followed by model optimization (to reduce the error between the model and the recorded CMAP scans) using the “Hunt” procedure. In this study, the default settings, including relative spread (RS) of motor unit threshold and motor unit size, were used at both research sites. The outputs of the program included MUNE, number of steps used in the calculation, error score, and motor unit size, etc. The MUNE values with percentage error < 7% were accepted as valid estimates (Bostock, 2016), and this was the case for all subjects in the present study. For each scan, the program was run three times including the setting of the prescan and postscan limits. The trial that gave the smallest error was accepted as the final estimate. In addition to MScanFit MUNE, D50, defined as the number of largest consecutive differences that are needed to build-up 50% of the maximum CMAP (Sleutjes et al., 2014), was also calculated from each CMAP scan.

### Statistical analysis

Comparisons of CMAP scan and MUNE variables were made under different stimulation protocols and at different research centers. A two-way repeated measures ANOVA was applied to evaluate whether features of the stimulation protocol (pulse width and number of steps) affected CMAP scan and modeled variables (i.e., MUNE). An unpaired *t*-test was performed to examine whether significant differences existed in CMAP scan and modeled variables between the research sites (UTHealth and SJTU). Statistical significance was set as *p* < 0.05. The analyses were performed using the SPSS software (SPSS, Chicago, IL).

## Results

### The effect of pulse duration and number of steps on compound muscle action potential scan parameters (UTHealth site)

**FIGURE 2:**
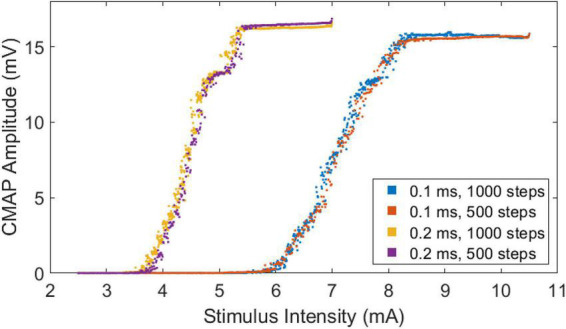
Stimulus-response curves of a representative subject based at four different CMAP scan settings.

All subjects completed the four CMAP scan protocols without incident. The recorded CMAP scan curves were sigmoid in shape regardless of differences in pulse width or number of steps. An example of CMAP scans from a representative subject is shown (Figure 2). The scans at different pulse widths display different patterns. Scans at a pulse width of 0.1 ms have a broader stimulation range and higher intensities compared with scans at a pulse width of 0.2 ms. In contrast, curves at different steps (500 or 1,000) demonstrate similar patterns as long as they have the same pulse width. It can be observed from the figure that the slopes of the scans at a pulse width of 0.2 ms are higher than the slopes at a pulse width of 0.1 ms.

Table 1 shows the lower (S0), upper (S100), and range (S100-S0) of stimulus thresholds at two different stimulus pulse widths for subjects from the UTHealth site, and the derived CMAP scan parameters from each stimulation protocol averaged across all subjects. It indicates that the lower/upper thresholds and threshold range were significantly lower at longer compared to shorter pulse widths. Pulse width had no significant effect on D50 (*p* > 0.1). As expected, D50 was significantly higher using 1,000 steps than 500 steps (*p* < 0.01). The CMAP amplitude was not affected by pulse width or the number of steps, and no significant interaction of pulse width and steps was found across four different protocols (*p* > 0.05).

**TABLE 1:** Parameters derived from CMAP scan of the APB using four different protocols (recorded at the UTHealth site, data in mean ± standard deviation).

Stimulus duration	0.1 ms		0.2 ms	
S0 (mA)	4.25 ± 1.21		2.65 ± 0.67	
S100 (mA)	12.70 ± 4.56		8.05 ± 3.40	
S100-S0 (mA)	8.45 ± 4.44		5.40 ± 2.95	

**Stimulus steps**	**500**	**1,000**	**500**	**1,000**

CMAP (mV)	14.67 ± 3.33	14.82 ± 3.53	15.45 ± 3.67	15.28 ± 4.11
D50	39.40 ± 7.52	67.10 ± 10.72	33.10 ± 12.38	62.10 ± 27.62
MScanFit MUNE	84.70 ± 21.56	77.90 ± 27.62	67.60 ± 18.72	62.20 ± 15.82
Mean unit size (μV)	178.78 ± 44.93	205.55 ± 66.91	242.72 ± 84.93	257.04 ± 85.55

MScanFit MUNE was significantly higher at the short compared to the long pulse widths in the 500 and 1,000 step protocols (*p* < 0.05). In contrast, the number of steps had no significant effect on MUNE (*p* > 0.1) (Table 1). No significant interaction of pulse width and steps was found using four different protocols (*p* > 0.5). Mean motor unit size was significantly lower at the short compared to the long pulse widths in the 500 and 1,000 step protocols (*p* < 0.05) (Table 1). In contrast, the number of steps had had no significant effect on mean motor unit size (*p* > 0.05). No significant interaction of pulse width and steps was found across four different protocols (*p* > 0.5).

### Comparison of UTHealth and SJTU sites

Mean CMAP scan parameters and MUNE, based on 0.1 ms pulse duration and 500 step protocol, for the two research sites are presented (Table 2). The lower threshold (S0) was significantly lower at the UTHealth site compared to the SJTU site, but the higher threshold (S100) and stimulus range (S100-S0) were similar between sites. The CMAP amplitude was slightly larger at the UTHealth site, but the difference was marginally significant. The MUNE was significantly higher in the SJTU than the UTHealth site, whereas D50 was similar.

**TABLE 2:** CMAP scan and MUNE parameters of the APB in two research centers using the same stimulus protocol (data in mean ± standard deviation).

Research sites	UTHealth (*n* = 10)	SJTU (*n* = 12)	
S0 (mA)	4.25 ± 1.21	6.21 ± 2.20	*p* = 0.02, *t* = 2.64
S100 (mA)	12.70 ± 4.56	15.54 ± 4.61	*p* = 0.16, *t* = 1.45
S100-S0 (mA)	8.45 ± 4.44	9.33 ± 2.89	*p* = 0.60, *t* = 0.54
CMAP(mV)	14.67 ± 3.33	12.01 ± 2.52	*p* = 0.05, *t* = 2.08
MScanFit MUNE	84.70 ± 21.56	136.42 ± 32.46	*p* < 0.001, *t* = 4.46
Mean MU size (μV)	178.78 ± 44.93	89.12 ± 19.05	*p* < 0.001, *t* = 6.29
D50	39.40 ± 7.52	38.83 ± 9.93	*p* = 0.88, *t* = 0.15

To rule out the effect of any unaware MScanFit program parameters on the result (that may cause the MUNE difference between the two sites), the UTHealth CMAP scan data was run again with the SJTU MScanFit software. The MUNE obtained was similar using the software in different sites with the same data (UTHealth: 84.7 ± 21.56; SJTU: 86.0 ± 24.57; *t* = 0.65, *p* > 0.5).

## Discussion

This study investigated the performance of MScanFit MUNE of the APB using different CMAP scan stimulation protocols at two research centers. Through testing of a combination of two stimulus pulse widths (0.1, 0.2 ms) and two steps (500, 1,000), it was found that MScanFit MUNE was significantly affected by pulse width but not the number of steps. This is consistent with findings in the FDI muscle (Zong et al., 2020). A shorter stimulus pulse duration allows for a higher resolution of the CMAP scan, as reflected by the lower stimulus-response slope, compared to a longer pulse duration. The slope of the stimulus-response curve is very important for refining the model in the MScanFit program (Bostock, 2016). This significant difference in MUNE at different pulse widths can be interpreted by the inverse linear relationship between the MUNE and the slope of the stimulus-response curve. On the other hand, at the same pulse width, slopes were similar for 500 and 1,000 steps. Consequently, MUNE was higher when recorded with 0.1 ms pulse width than 0.2 ms pulse width, whereas MUNE was unrelated to the number of stimuli. A further reduction in stimulus pulse width (for example, to 0.05 ms) may continue to increase the resolution of CMAP scan and decease the slope of the stimulus-response curve. However, this would require higher stimulus intensity to activate all motor units, that may be beyond the maximal current output of the stimulator. The use of short pulse durations is particularly problematic in certain patient populations that have high axonal thresholds (Nodera et al., 2004; Henderson et al., 2006). It may also a problem for examination of the nerves that require higher stimulus intensities. Although this study examined pulse duration of 0.1 ms and 0.2 ms, standardized longer pulse duration might be necessary for such nerves. For example, a pulse duration of 0.5 or 1 ms was used for CMAP scan examination of tibialis anterior and peroneus longus muscles innervated by peroneal nerve (Kristensen et al., 2020; Sørensen et al., 2022; Tankisi et al., 2022). Given that there was no significant difference in MUNE between protocols of 500 and 1,000 steps at the same pulse width, the protocol of 500 steps is preferred because it requires less time to record and is thus more tolerable for the subject.

An unexpected finding of this study was the significantly larger MUNE at the SJTU than the UTHealth site, even though the same pulse width (0.1 ms) and steps (500) were applied at each site. MScanFit has also been used by others to estimate the number of APB motor units from healthy control subjects based on CMAP scan recordings (Jacobsen et al., 2017, 2018b; Sirin et al., 2019; Higashihara et al., 2020; Schneider et al., 2021). Interestingly, MUNE derived at the SJTU and UTHealth sites was close to the high and low ends of the previously reported results. It should be emphasized that although our research was performed in two countries, examination of ethnicity-based difference was not intended. Indeed, a majority of subjects in the UTHealth site were Asian ethnicity. Given this, ethnicity-based difference is not likely to be a factor. This also applies to aging since both groups of subjects are young adults. In both UTHealth and SJTU sites, the data collection was carried out by the same experimenter (YZ—who worked as visiting scientist in UTHealth), with help of others. Therefore, the experimenter factor can also be excluded.

The difference may be partially explained by different electrode size in the two centers. There are different factors that may collectively contribute to the CMAP amplitude. For example, on one hand, a larger electrode can record larger volume of the muscle which should result in a larger CMAP amplitude. On the other hand, the action potential recorded by an electrode is the average of the potentials over the recording surface. This provides a low pass filtering effect thus reducing the action potential amplitude. The inverse relation between CMAP amplitude and surface electrode size was reported in previous studies (Wee and Ashley, 1990; Barkhaus et al., 2006), i.e., the CMAP amplitude decreased with increasing electrode size, whereas waveform shape was relatively well preserved, and this effect was most pronounced in small muscles. In the current study, the recording electrode was larger at the SJTU than the UTHealth site, and may partly account for the smaller CMAP amplitude. We found that there was no significant difference in stimulus intensity range between the two sites. Therefore, the slope of the stimulus-response curve tended to be higher in the UTHealth site compared with the SJTU site. As discussed earlier, the CMAP scan slope is closely related to MScanFit MUNE. The significant lower MUNE at the UTHealth site can be partly interpreted by the inverse relationship between the MScanFit MUNE and the slope of the stimulus-response curve. It remains to be determined whether there are other factors that may collectively contribute to the significant different MUNE values.

The current study is limited by only examining the APB muscle of a small number of neurologically intact subjects, and lack of a test-retest analysis. Only two different pulse widths and steps were examined. The findings of the study indicate the importance of applying consistent CMAP scan experimental parameters when comparing or tracking MScanFit MUNE changes. To further confirm or examine the effect of electrode size on CMAP amplitude and MScanFit MUNE, our future work will involve testing the same subjects with different electrode sizes.

## Data availability statement

The raw data supporting the conclusions of this article will be made available by the authors, without undue reservation.

## Ethics statement

The studies involving human participants were reviewed and approved by the Committee for Protection of Human Subjects (CPHS) at University of Texas Health Science Center at Houston (UTHealth) and TIRR Memorial Hermann Hospital (Houston, TX), and the Institutional Review Board of Shanghai Jiao Tong University. The patients/participants provided their written informed consent to participate in this study.

## Author contributions

PZ, QX, SL, and YZh: study design. YZo, PX, and ZL: data collection. YZo, ZL, MC, LD, SL, YZh, QX, and PZ: data analysis and interpretation. YZo and ZL: writing—original draft preparation. PZ: writing—revision. MC, PX, LD, SL, YZh, and QX: writing—review and editing. QX and PZ: study supervision. All authors have read and agreed to the published version of the manuscript.

## References

[B1] Barkhaus P. E. Periquet M. I. Nandedkar S. D. (2006). Influence of the surface EMG electrode on the compound muscle action potential. *Electromyogr. Clin. Neurophysiol.* 46 235–239. 16929630

[B2] Bostock H. (2016). Estimating motor unit numbers from a CMAP scan. *Muscle Nerve.* 53 889–896. 10.1002/mus.24945 26479267

[B3] de Carvalho M. Barkhaus P. E. Nandedkar S. D. Swash M. (2018). Motor unit number estimation (MUNE): Where are we now? *Clin. Neurophysiol.* 129 1507–1516. 10.1016/j.clinph.2018.04.748 29804042

[B4] Henderson R. D. Ridall G. R. PettittA N. McCombe P. A. Daube J. R. (2006). The stimulus-response curve and motor unit variability in normal subjects and subjects with amyotrophic lateral sclerosis. *Muscle Nerve* 34 34–43. 10.1002/mus.20561 16634059

[B5] Higashihara M. Menon P. van den Bos M. Pavey N. Vucic S. (2020). Reproducibility of motor unit number index and MScanFit motor unit number estimation across intrinsic hand muscles. *Muscle Nerve* 62 192–200. 10.1002/mus.26839 32077117

[B6] Jacobsen A. B. Bostock H. Tankisi H. (2018a). CMAP Scan MUNE (MScan) - A novel motor unit number estimation (MUNE) method. *J. Vis. Exp.* 7:56805. 10.3791/56805 29939177 PMC6101587

[B7] Jacobsen A. B. Bostock H. Fuglsang-Frederiksen A. Duez L. Beniczky S. Møller A. T. (2017). Reproducibility, and sensitivity to motor unit loss in amyotrophic lateral sclerosis, of a novel MUNE method: MScanFit MUNE. *Clin. Neurophysiol.* 128 1380–1388. 10.1016/j.clinph.2017.03.045 28461135

[B8] Jacobsen A. B. Kristensen R. S. Witt A. Kristensen A. G. Duez L. Beniczky S. (2018b). The utility of motor unit number estimation methods versus quantitative motor unit potential analysis in diagnosis of ALS. *Clin. Neurophysiol.* 129 646–653. 10.1016/j.clinph.2018.01.002 29414408

[B9] Kristensen A. G. Khan K. S. Bostock H. Khan B. S. Gylfadottir S. Andersen H. (2020). MScanFit motor unit number estimation and muscle velocity recovery cycle recordings in diabetic polyneuropathy. *Clin. Neurophysiol.* 131 2591–2599. 10.1016/j.clinph.2020.07.017 32927215

[B10] Maathuis E. M. Henderson R. D. Drenthen J. Hutchinson N. M. Daube J. R. Blok J. H. (2012). Optimal stimulation settings for CMAP scan registrations. *J. Brachial Plex. Peripher. Nerve Inj.* 7:4. 22546084 10.1186/1749-7221-7-4 PMC3377546

[B11] Nodera H. Bostock H. Kuwabara S. Sakamoto T. Asanuma K. Jia-Ying S. (2004). Nerve excitability properties in charcotmarie-tooth disease type 1A. *Brain* 127 203–211. 10.1093/brain/awh020 14607794

[B12] Schneider C. Wassermann M. K. Grether N. B. Fink G. R. Wunderlich G. Lehmann H. C. (2021). Motor unit number estimation in adult patients with spinal muscular atrophy treated with nusinersen. *Eur. J. Neurol.* 28 3022–3029. 10.1111/ene.15005 34216082

[B13] Sirin N. G. Oguz Akarsu E. Kocasoy Orhan E. Erbas B. Artug T. Dede H. O. (2019). Parameters derived from compound muscle action potential scan for discriminating amyotrophic lateral sclerosis-related denervation. *Muscle Nerve* 60 400–408. 10.1002/mus.26644 31330055

[B14] Sleutjes B. T. H. M. Ruisch J. Nassi T. E. Buitenweg J. R. van Schelven L. J. van den Berg L. H. (2021). Impact of stimulus duration on motor unit thresholds and alternation in compound muscle action potential scans. *Clin. Neurophysiol.* 132 323–331. 10.1016/j.clinph.2020.10.026 33450554

[B15] Sleutjes B. Montfoort I. Maathuis E. M. Drenthen J. van Doorn P. A. Visser G. H. (2014). CMAP scan discontinuities: Automated detection and relation to motor unit loss. *Clin. Neurophysiol.* 125 388–395. 10.1016/j.clinph.2013.07.016 23993681

[B16] Sørensen D. M. Bostock H. Ballegaard M. Fuglsang-Frederiksen A. Graffe C. C. Grötting A. (2022). Assessing inter-rater reproducibility in MScanFit MUNE in a 6-subject, 12-rater “Round Robin” setup. *Neurophysiol. Clin.* 52 157–169. 10.1016/j.neucli.2021.11.002 34906430

[B17] Tankisi D. A. Alaydin H. C. Boran E. Cengiz B. (2022). Feasibility and reliability of MScanFit motor unit number estimation in peroneus longus muscle. *Muscle Nerve* 66 503–507. 10.1002/mus.27667 35763284

[B18] Visser G. H. Blok J. H. (2009). The CMAP scan. *Suppl. Clin. Neurophysiol.* 60 65–77. 10.1016/S1567-424X(08)00006-8 20715368

[B19] Wee A. S. Ashley R. A. (1990). Relationship between the size of the recording electrodes and morphology of the compound muscle action potentials. *Electromyogr. Clin. Neurophysiol.* 30 165–168. 2351092

[B20] Zong Y. Lu Z. Zhang L. Li X. Zhou P. (2020). Motor unit number of the first dorsal interosseous muscle estimated from CMAP scan with different pulse widths and steps. *J. Neural. Eng.* 17:014001. 10.1088/1741-2552/ab57cc 31726441

